# “Hearing the Ovarian Cancer Whisper”: A Pipeline Program Introducing Medical Students to Gynecologic Oncology, Focusing on the Diagnosis and Management of Ovarian Cancer

**DOI:** 10.1007/s40670-026-02904-3

**Published:** 2026-09-29

**Authors:** Angela Slaybe, Noël C. Barengo, Carolyn D. Runowicz

**Affiliations:** Herbert Wertheim College of Medicine, Florida International University, Miami, FL USA

## Abstract

**Purpose:**

Pipeline programs help medical students explore and define future career pathways. The “Hearing the Ovarian Cancer Whisper” (HOW) Dr. Robert C. Knapp Award was created to expose medical students to gynecologic oncology, with emphasis on recognizing, treating, and improving outcomes for patients with ovarian cancer. This study describes and analyzes the characteristics of HOW participants and their eventual residency choices.

**Method:**

We conducted a non-concurrent cohort study evaluation using the HOW database of medical students who received the Dr. Robert C. Knapp Medical Student Award and completed the four-week gynecologic oncology elective between 2011 and 2025. Baseline information included medical school, sex, and scholarship year. Follow-up information was obtained through phone, email, or text communication and included residency, fellowship, specialty, and practice location. Of 149 award recipients, 24 were excluded because of missing information, nonresponse, dropout, or pending follow-up, resulting in 125 participants in the primary analytic cohort.

**Results:**

Among the 125 participants included in the primary analysis, 53.6% entered an OBGYN residency and 46.4% another specialty. Three participants subsequently pursued gynecologic oncology fellowship training during the observation period. Most participants were female (84.0%). Florida International University Herbert Wertheim College of Medicine accounted for the largest proportion of participants (44.0%). The distribution of OBGYN residency entry varied by scholarship year, although the overall association did not reach statistical significance (*p* = 0.080).

**Conclusion:**

These findings describe the longitudinal career trajectories of participants in a gynecologic oncology educational program but do not establish that program participation caused or independently influenced specialty choice.

## Introduction

Hearing the Ovarian Cancer Whisper (HOW) is a Florida-based 501(c)(3) charitable organization dedicated to increasing awareness of ovarian cancer and supporting educational, clinical, and research activities related to gynecologic oncology. The Dr. Robert C. Knapp Medical Student Award was established in 2011 to provide medical students with support for a four-week elective in gynecologic oncology under the supervision and mentorship of a gynecologic oncologist. Selection criteria of the award include selection of a program with a gynecologic oncologist by a fourth-year medical student and a formal program description by the applicant. The educational goals of the program include increasing students’ exposure to gynecologic oncology and improving their understanding of the presentation, diagnosis, and treatment of ovarian cancer. It is important for future physicians to know about the subspecialty of gynecologic oncology to appropriately refer patients suspected of ovarian cancer to a gynecologic oncologist for the best outcomes [1]. This project evaluated the residency choice of HOW recipients from 2011 to 2025 and for those who completed residency, the location and type of practice/specialty/fellowship.

There are many pipeline programs that focus on oncology in general, which include the American Society of Clinical Oncology. There are also more specific but limited programs in gynecologic oncology through the Society of Gynecologic Oncology and the Memorial Sloan Kettering Cancer Center. These opportunities, like HOW, allow medical students to elect clinical experiences in gynecologic oncology. However, the outcomes of these pipeline programs have not been published. HOW’s objective with the Dr. Robert C. Knapp award was to bring a greater awareness of the signs, symptoms, and treatment of ovarian cancer to Florida medical students. This goal of bringing awareness to a surgical specialty is similar to a program by the Nth Dimensions Summer Internship which sought to recruit more women into surgical and procedural specialties [2]. However, unlike HOW, the Nth Dimensions Summer Internship program’s goal was in recruitment while HOW focuses more on raising awareness of ovarian cancer and knowledge about the subspecialty of gynecologic oncology. The publications on oncology pipeline programs analyze the impact they have on students in terms of emphasizing the importance of strategic partnerships, career development pathways, and local talent pipelines in building a sustainable and diverse oncology workforce [3]. However, these papers do not investigate the specific outcomes of the individual students who participate in these oncologic pipeline programs.

In addition, pipeline programs allow for opportunities for mentorship in undergraduate medical education [4]. Mentorship in medical education has been shown to improve student career outcomes, highlighting the importance of pipeline programs especially in the current competitive nature of the residency application and match process [5]. Ultimately, it is important to review outcomes of these pipeline programs in order to have an improved knowledge of how to best aid medical students in their career development.

The primary research question was: Among students who received the HOW Dr. Robert C. Knapp Medical Student Award and completed the associated elective, what proportion subsequently entered an OBGYN residency? Secondary objectives were to describe participants’ demographic and medical school characteristics and, among participants with available follow-up, their subsequent fellowship, specialty, and geographic practice outcome.

## Materials and Methods

### Study Design and Participants

This study was a non-concurrent cohort study using the HOW database which included awardees of the Dr. Robert C. Knapp Medical Student award from 2011 to 2025.

**Fig. 1:**
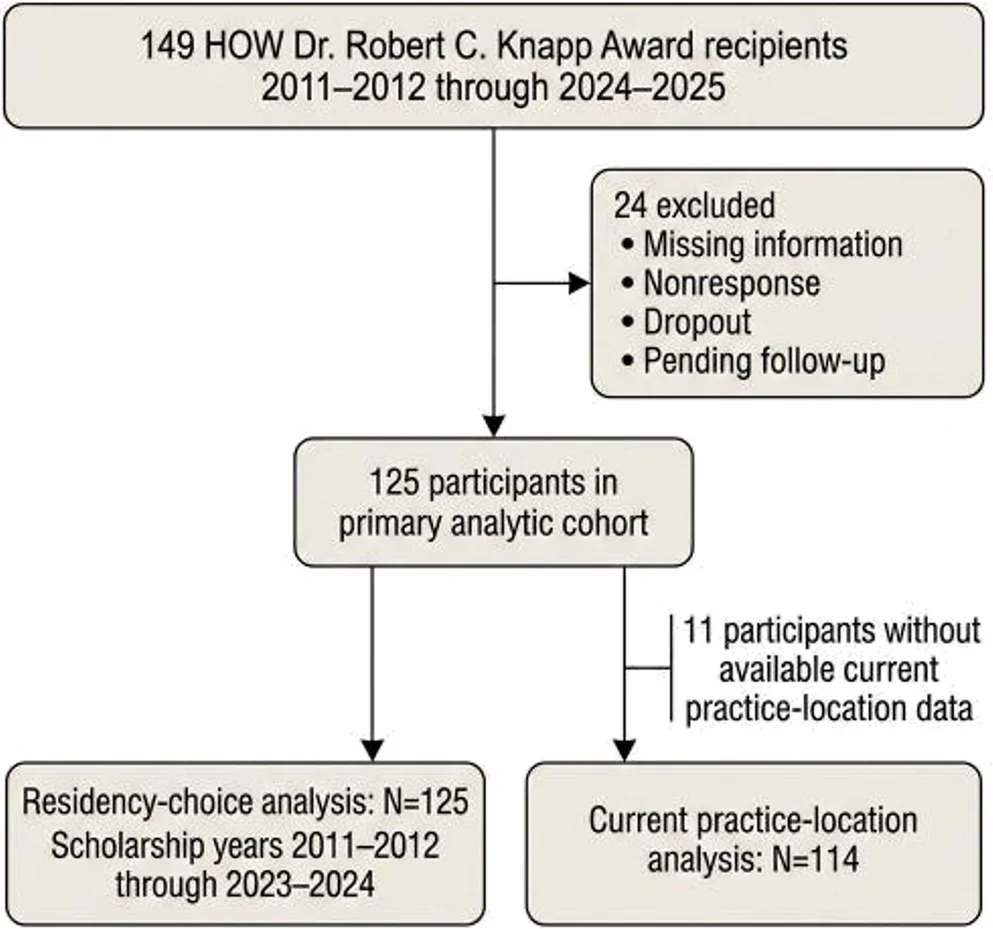
Derivation of the analytic samples

The database of medical student recipients of the Dr. Robert C. Knapp award from 2011 to 2025 was reviewed and analyzed. The students were selected for the award based on a review of their application by staff at HOW and a gynecologic oncologist. The applicant identified a gynecologic oncologist as their mentor for a four-week elective in gynecologic oncology. Preference was given to students from Florida medical schools, however, medical students from other states were included notably in the earlier years of the pipeline program. Inclusion criteria were male and female medical students who participated in a four-week elective under the supervision of a gynecologic oncologist from 2011 to 2025 (*n* = 149). Participants with missing information and data from participants that did not answer, dropped out, or have pending responses were excluded from the analysis (*n* = 24). The final sample analyzed included 125 awardees (Fig. 1) for the analysis presented in Tables 1 and 2. As 11 participants did not have information on the region they are currently practicing, the total of participants for the analysis in Table 3 was *N* = 114.

**Table 1:** Characteristics of HOW Dr. Robert C. Knapp award recipients included in the primary analytic cohort, 2011–2025 (*N* = 125)

	% (95% CI)	(*n*)
**Sex (** ***N*** ** = 125)**		
Male	16.0 (10.1–23.6)	(20)
Female	84.0 (76.4–89.9)	(105)
**Medical School Location (** ***N*** ** = 149)**		
Florida International University	44.0 (35.1–53.2)	(55)
University of Central Florida	9.6 (5.1–16.2)	(12)
University of Florida	1.6 (0.2–5.7)	(2)
University of Miami	24.0 (16.8–32.5)	(30)
University of South Florida	5.6 (2.3–11.2)	(7)
Florida State University	4.0 (1.3–9.1)	(5)
Florida Atlantic University	0.8 (0.0-4.4)	(1)
Duke University	2.4 (0.5–6.9)	(3)
Advent Health (Florida)	0.8 (0.0-4.4)	(1)
Weil Cornell	5.6 (2.3–11.2)	(7)
Lake Erie College of Osteopathic Medicine	0.8 (0.0 -4.4)	(1)

The primary analytic cohort included 125 of 149 award recipients. Twenty-four recipients were excluded because of missing information, nonresponse, dropout, or pending follow-up. Percentages are calculated using participants with available data (*N* = 125) as the denominator. Percentages may not total 100% because of rounding

**Table 2:** OBGYN residency entry by scholarship year among HOW award recipients with available residency-choice data, 2011–2012 through 2023–2024 (*N* = 125)

	Entered OBGYN residency program		
	Yes	No	*p*-value
*N* = 125	% (*n*)	% (*n*)	
**Scholarship year**			0.065
2023–2024	66.7 (4)	33.3 (2)	
2022–2023	72.2 (8)	27.3 (3)	
2021–2022	100.0 (8)	0.0 (0)	
2020–2021	75.0 (6)	25.0 (2)	
2019–2020	50.0 (5)	50.0 (5)	
2018–2019	50.0 (5)	50.0 (5)	
2017–2018	75.0 (6)	25.0 (2)	
2016–2017	33.3 (4)	66.7 (8)	
2015–2016	30.0 (3)	70.0 (7)	
2014–2015	41.7 (5)	58.3 (7)	
2013–2014	40.0 (6)	60.0 (9)	
2012–2013	54.5 (6)	45.5 (5)	
2011–2012	25.0 (1)	75.0 (3)	
*Total*	*53.6 (67)*	*46.4 (58)*	

Participants from the 2024–2025 scholarship year were not included in the residency-choice analysis because they had not yet selected a residency at the time of data collection. Percentages are calculated within each scholarship year. The association between scholarship year and OBGYN residency entry was evaluated using Fisher–Freeman–Halton exact testing because of small cell counts

**Table 3:** Region of current medical practice among HOW award recipients with available practice-location data (*N* = 114)

	% (95% CI)	(*n*)
**Region of Current Medical Practice (** ***N*** ** = 114)**		
Northeast	27.2 (19.3–36.3)	(31)
Southeast	44.7 (35.4–54.3)	(51)
Northwest	4.4 (1.4–9.9)	(5)
Southwest	5.3 (2.0-11.1)	(6)
Central	12.3 (6.9–19.7)	(14)
West	6.1 (2.5–12.2)	(7)

Current practice location was available for 114 participants. Percentages are calculated using *N* = 114 as the denominator

### Variables

The main outcome of the study was the number of students who chose residencies in obstetrics and gynecology. The student characteristics assessed were name of medical school, sex, year of residency completion, scholarship year, career choice in gynecologic oncology and location of current practice. There were 11 medical schools: Florida International University Herbert Wertheim College of Medicine, University of Central Florida College of Medicine, University of Miami Miller School of Medicine, University of South Florida Morsani School of Medicine, Florida State University College of Medicine Florida Atlantic University Charles E. Schmidt College of Medicine, Advent Health, and four non-Florida medical schools: Weil Cornell Medical College, Brigham and Women’s Hospital, Lake Erie College of Osteopathic Medicine, and Duke University School of Medicine. Year of residency completion ranged from 2016 to 2028. Sex was dichotomized into male versus female. Scholarship year ranged from 2011–2012 to 2024–2025. Whether individuals pursued a career in gynecologic oncology was dichotomized into yes or no, where pursuing a career in gynecologic oncology is the outcome variable. If individuals did pursue a career in gynecologic oncology, the location of their current practice was dichotomized into practicing in the state of Florida or outside the state of Florida. If individuals did not pursue a career in gynecologic oncology, the type of medicine they are practicing was categorized as: general OBGYN, Maternal-Fetal Medicine, Reproductive Endocrinology, Complex Family Planning, Pediatrics, Pediatric Emergency Medicine, Emergency Medicine, General Surgery, Breast Surgery, Orthopedic Surgery, Gynecologic Surgery, Plastic Surgery, Surgical Science, Urology, Post-doctoral fellow in cancer biology in department of neurosurgery, Anesthesiology, Family Medicine, Internal Medicine, Radiation Oncology, Women’s Imaging, Abdominal Imaging, Breast Radiology, Diagnostic Radiology, Medical Oncology (prostate cancer), Cardiovascular Disease, Psychiatry, and Dermatology. If individuals are not practicing gynecologic oncology, the location of their current practice was recorded as Florida, Massachusetts, Tennessee, Alabama, New Jersey, Ohio, Louisiana, New York, Connecticut, Illinois, Texas, Colorado, Pennsylvania, Georgia, Washington, Virginia, Kentucky, Arizona, Maryland, Minnesota, North Carolina, California, Washington D.C., Rhode Island, Nebraska, Hawaii, Oregon, and New Mexico. Of note, at the end of the awardees’ four-week rotation, a report was required to be submitted by each awardee which included the experience/knowledge gained by the students in the diagnosis and treatment of women with ovarian cancer.

### Statistical Analysis

Data were analyzed using IBM SPSS Statistics version 29.0.2.0. Participant characteristics and outcomes were summarized using absolute frequencies and percentages. Exact binomial 95% confidence intervals (Clopper–Pearson method) were calculated for key descriptive proportions. The association between scholarship year and subsequent entry into an OBGYN residency was evaluated using Fisher–Freeman–Halton exact test/Monte Carlo exact test because several cells had small expected frequencies, making the assumptions of the Pearson chi-square test inappropriate. All tests were two-sided, and p-values < 0.05 were considered statistically significant. Analyses were conducted using available data for each outcome. Of the 149 award recipients, 24 were excluded from the primary analytic cohort because of missing information, nonresponse, dropout, or pending follow-up, resulting in 125 participants for analyses of participant characteristics and residency choice. For current practice region, information was available for 114 participants; percentages for this outcome were therefore calculated using 114 as the denominator. No imputation of missing data were performed.

### Ethical Considerations

The study protocol was approved by the Institutional Review Board (IRB) of the Florida International University Herbert Wertheim College of Medicine.

## Results

A total of 149 students received the Dr. Robert C. Knapp Medical Student Award between the 2011–2012 and 2024–2025 scholarship years. Of these, 24 were excluded because of missing information, nonresponse, dropout, or pending follow-up, leaving 125 participants in the primary analytic cohort. Participant characteristics for these 125 awardees are presented in Table 1. Among the 125 participants with available residency-choice data from the 2011–2012 through 2023–2024 scholarship years, 67 (53.6%) entered an OBGYN residency and 58 (46.4%) entered another specialty. Participants from the 2024–2025 scholarship year were not included in the residency-choice analysis because they had not yet selected a residency at the time of data collection. Information on current geographic practice location was available for 114 participants and is presented separately in Table 3. Among the 125 participants in the primary analytic cohort, 105 (84.0%) were female and 20 (16.0%) were male. Florida International University Herbert Wertheim College of Medicine accounted for the largest proportion of participants (55/125; 44.0%). Additional participant characteristics are presented in Table 1. Among the 125 participants with residency-choice information, 67 (53.6%) entered an OBGYN residency and 58 (46.4%) entered another specialty. The proportion entering OBGYN varied across scholarship years, ranging from 25.0% in 2011–2012 to 100.0% in 2021–2022 (Table 2). Because several scholarship-year categories contained small cell counts, the association between scholarship year and OBGYN residency entry was evaluated using Fisher–Freeman–Halton exact testing. The p-value of the association was not statistically significant (p-value: 0.065).

Current practice location was available for 114 participants. Among these participants, 51/114 (44.7%; 95% CI 35.4–54.3) were practicing in the Southeast, 31/114 (27.2%; 95% CI 19.3–36.3) in the Northeast, 14/114 (12.3%; 95% CI 6.9–19.7) in the Central region, 7/114 (6.1%; 95% CI 2.5–12.2) in the West, 6/114 (5.3%; 95% CI 2.0–11.1) in the Southwest, and 5/114 (4.4%; 95% CI 1.4–9.9) in the Northwest (Table 3).

## Discussion and Conclusions

The HOW medical student program provided participants with structured exposure to gynecologic oncology and ovarian cancer care. More than half (53.6%) of participants with available residency-choice data subsequently entered OBGYN residency. However, given the observational design and absence of a comparison group, this finding should be interpreted as a description of participants’ subsequent career trajectories rather than evidence that program participation influenced specialty choice. This program introduced medical students to gynecologic oncology as a subspecialty and importantly educated them about the diagnosis and treatment of women with ovarian cancer. The goal of the program was not for them to become gynecologic oncologists, but to enhance their experience in treating patients with ovarian cancer. Most recipients of the award, 84.0%, were females. This was not unexpected as national statistics demonstrate that most medical students entering OBGYN are female [6]. As our data suggest, the majority of the award recipients selected residencies in OBGYN. Of note, there were also residents who subspecialized in maternal fetal medicine, reproductive endocrinology, gynecologic surgery, complex family planning, and women’s imaging.

Analysis of the overall data demonstrated that only 53.6% of awardees from 2011 to 2024 pursued careers in OBGYN. The proportion of awardees entering OBGYN residency varied across scholarship years, with higher proportions observed in several years from 2017 to 2018 onward: 75.0% in 2017–2018, 50.0% in 2018–2019, 50.0% in 2019–2020, 75.0% in 2020–2021, 100.0% in 2021–2022, 72.2% in 2022–2023, and 66.7% in 2023–2024. Although these later scholarship years generally demonstrated higher proportions of OBGYN residency entry compared with several earlier years, the small number of participants in each scholarship year limits interpretation of this pattern. The apparent increase during several later scholarship years, including the period encompassing the COVID-19 pandemic, is of potential interest. However, the association between scholarship year and OBGYN residency entry was not statistically significant (*p* = 0.080), and the small number of participants in individual years limits the reliability of year-specific comparisons. The observed variation may reflect changes in the applicant pool, students’ pre-existing specialty interests, or changes in the competitiveness of OBGYN residency programs [7] rather than an effect of participation in the HOW program.

HOW’s objective for the Dr. Robert C. Knapp award was to bring a greater awareness of the presenting signs, symptoms, and treatment of patients with ovarian cancer to Florida medical students. It is important to note that the majority of HOW awardees were from Florida International University Herbert Wertheim College of Medicine. This is mainly due to the involvement of Carolyn D. Runowicz MD, who is a current board member of HOW and is a professor emeritus at Florida International University Herbert Wertheim College of Medicine and actively supported and promoted this program to the Herbert Wertheim College of Medical School students. Her engagement demonstrated that having a “champion” of a pipeline program is an effective strategy in recruiting students.

Review of the literature for pipeline programs in oncology analyzes the impact they have on students in terms of emphasizing the importance of career development pathways, and local talent pipelines in building a sustainable and diverse oncology workforce [3]. However, these papers do not track the specific outcomes of the individual students who participate in these oncology pipeline programs. A study in radiation oncology analyzed the success of pipeline programs in reaching medical students and introducing them to radiation oncology, with an objective to increase exposure of this specialty to medical students throughout the country [8]. Another study by Ben-David et al. assessed the impact of general oncology pipeline programs earlier in medical school education [9]. This study highlighted the importance of mentorship and research experience for medical students pursuing a career in oncology through a summer research experience at the Tisch Cancer Institute at Mount Sinai, New York. Another study analyzed the outcomes of fourth year medical students going into OBGYN residencies and found that many medical students who are incoming OBGYN residents have limited exposure to this specialty [10]. This study is of note as one of the objectives of the HOW Dr. Robert C. Knapp Award is to expose future physicians to the specialty of gynecologic oncology to increase their knowledge of ovarian cancer presentation and treatment by gynecologic oncologists. Such exposure may provide students with additional information and experience relevant to subsequent specialty decisions; however, the present study was not designed to determine whether participation influenced those decisions.

HOW’s ability to increase student awareness in the surgical specialty of gynecologic oncology is of note, as medical students may have limited exposure of medical specialties [11] depending on their curriculum and available rotation sites. This underscores the importance of pipeline programs that allow further exposure to specialties outside students’ medical education, thus allowing for a more educated decision in the residency selection process. The observed proportion of participants subsequently entering OBGYN suggests that programs such as HOW may reach medical students with an interest in OBGYN and related fields. Whether participation itself influences subsequent specialty choice cannot be determined from the present study. In addition, medical student choice in selecting which specialty to practice has been shown to be influenced by many aspects which include “clinical-practical experience”, “clinical role models”, and “voluntary offerings” [12]. Allowing medical students to play an active role in their education, such as the opportunity presented by participating in the HOW pipeline program allows further exposure and involvement in OBGYN as a specialty, increasing the potential for future interest.

This study has several important limitations. First, the non-concurrent cohort study design and absence of a comparison group prevent causal inference regarding the relationship between program participation and subsequent specialty choice. Second, participants self-selected into the application process, creating the potential for selection bias; students already interested in OBGYN, oncology, surgery, or women’s health may have been more likely to apply. Third, baseline interest in OBGYN and gynecologic oncology was not measured, preventing assessment of changes in career interest attributable to the program. Fourth, follow-up was incomplete for some participants, particularly those who had not yet completed residency or had not responded to follow-up requests. Fifth, several subgroup categories contained small numbers, limiting statistical power and making comparisons between individual years or specialties unstable. Sixth, the database was updated over time, resulting in different denominators for different outcomes. These denominators should therefore be explicitly reported for each analysis. Finally, although ovarian cancer knowledge was a central educational objective of the program, the study did not directly measure knowledge gain using a standardized pre- and post-program assessment.

In conclusion, the HOW Dr. Robert C. Knapp Medical Student Award provided medical students with structured exposure to gynecologic oncology and ovarian cancer care. Among participants with available follow-up, more than half subsequently entered OBGYN residency, and three pursued gynecologic oncology fellowship training. These findings describe the career trajectories of program participants but should not be interpreted as evidence that the program caused or increased entry into OBGYN, particularly given the absence of a comparison group, potential self-selection of participants, and lack of information on baseline specialty interest. Nevertheless, the findings highlight the feasibility of longitudinally tracking participants in specialty-focused educational programs and suggest several areas for future prospective evaluation, including changes in knowledge and specialty interest, the role of mentorship and local faculty champions, and comparisons with nonparticipating students.
